# Microalgal Biomass as Feedstock for Bacterial Production of PHA: Advances and Future Prospects

**DOI:** 10.3389/fbioe.2022.879476

**Published:** 2022-05-12

**Authors:** Florence Hui Ping Tan, Najiah Nadir, Kumar Sudesh

**Affiliations:** ^1^ School of Biological Sciences, Universiti Sains Malaysia, Penang, Malaysia; ^2^ PETRONAS Research Sdn. Bhd., Selangor, Malaysia

**Keywords:** microalgae, biomass, microalgal biomass production, polyhydroxyalkanoates (PHA), microbial PHA synthesis, photoautotrophy

## Abstract

The search for biodegradable plastics has become the focus in combating the global plastic pollution crisis. Polyhydroxyalkanoates (PHAs) are renewable substitutes to petroleum-based plastics with the ability to completely mineralize in soil, compost, and marine environments. The preferred choice of PHA synthesis is from bacteria or archaea. However, microbial production of PHAs faces a major drawback due to high production costs attributed to the high price of organic substrates as compared to synthetic plastics. As such, microalgal biomass presents a low-cost solution as feedstock for PHA synthesis. Photoautotrophic microalgae are ubiquitous in our ecosystem and thrive from utilizing easily accessible light, carbon dioxide and inorganic nutrients. Biomass production from microalgae offers advantages that include high yields, effective carbon dioxide capture, efficient treatment of effluents and the usage of infertile land. Nevertheless, the success of large-scale PHA synthesis using microalgal biomass faces constraints that encompass the entire flow of the microalgal biomass production, i.e., from molecular aspects of the microalgae to cultivation conditions to harvesting and drying microalgal biomass along with the conversion of the biomass into PHA. This review discusses approaches such as optimization of growth conditions, improvement of the microalgal biomass manufacturing technologies as well as the genetic engineering of both microalgae and PHA-producing bacteria with the purpose of refining PHA production from microalgal biomass.

## 1 Introduction

The universal plastic pollution emergency is edging towards an alarming irreversible “tipping point”. In 2019, approximately 370 million tons of plastic were produced worldwide, marking the highest expansion rate since the introduction of plastics for everyday use in the 1900s (Group, 2020). Owing to their high stability, synthetic petroleum-based plastics resist degradation, and inadvertently remain in the ecosystem for hundreds to thousands of years to come (Barnes et al., 2009). A projected annual flux of 4.8–12.7 million metric tons of plastic waste are discharged to ocean bodies (Jambeck et al., 2015), causing adverse injury to wildlife (Marn et al., 2020), damage to ecosystems (Lamb et al., 2018) and also harming human health (Thompson et al., 2009). In light of the recent COVID-19 pandemic, global plastic pollution has seen a surge in numbers with increased use of single-use plastics including personal protective equipment (Silva et al., 2021). In efforts to curb this predicament, biosynthetic and biodegradable plastics were introduced to the mass public.

Among the biodegradable polymers available, polyhydroxyalkanoates (PHAs) are suitable substitutes for some conventional plastics that offer unique benefits. Not only do these polymers possess similar mechanical properties to petroleum-based plastics such as polypropylene (PP), but they are also the only polymer that is 100% biodegradable (Khanna and Srivastava, 2005). PHAs are produced through microbial fermentation wherein carbon sources are metabolized into PHA and aggregate intracellularly in granules (Yu et al., 2006; Mathuriya et al., 2017). Over 300 species of microbes including Gram-negative and Gram-positive bacteria along with archaea and algae were reported to have PHA synthesizing capabilities (Grigore et al., 2019). The most commonly employed bacteria for PHA production are *Cupriavidus necator* (Sohn et al., 2021), *Bacillus* (Mohapatra et al., 2017) recombinant *Escherichia coli* (Leong et al., 2014), and *Pseudomonas* (Mozejko-Ciesielska et al., 2019)*.*

However, the large-scale application of PHA is plagued by high market prices compared to conventional plastics. The current commercial production of PHA costs up to €2.2 to 5.0 per kg, while conventional PP costs only €1.0 per kg (Liu et al., 2021). Such a huge difference is attributed to the production cost wherein PHA synthesis employs pure cultures and expensive carbon substrates while conventional plastics is economical due to the larger capacity of manufacturing a broad range of applications (Ong et al., 2018). Substrate prices account for 30–50% of the overall PHA cost (Choi and Lee, 1997). Thus, the search for suitable candidates for cost-effective feedstock that have high efficiency and high yield is necessary to ensure the success of PHA production on an industrial scale.

With high potential as feedstock biofactories, recent research has focused on the use of microalgal biomass as carbon source for PHA production because of the high carbohydrate yield and the lack of lignin which facilitates low-cost retrieval of fermentable sugars (Ghosh et al., 2019). Microalgae encompass a wide range of unicellular photosynthetic microorganisms and are ubiquitously found in all aquatic environments including freshwater and saltwater bodies with adapted tolerance to a wide range of abiotic and biotic stress (Rani et al., 2021). In terms of metabolism, microalgae are not only photoautotrophic but can adapt to heterotrophy or even mixotrophy depending on the environment (Mitra et al., 2012). While microalgae’s photosynthetic mechanism is analogous to that of terrestrial plants, the presence of pyrenoids for carbon dioxide fixation (Machingura and Moroney, 2018) in addition to aqueous habitats that enable easy access to growth requirements allow microalgae to yield biomass with efficiencies of at least two magnitudes higher than customary agricultural generation (Packer, 2009; Weyer et al., 2010). PHA production directly from microalgae has also been researched but the yield remains low. As such, the proposed “two module system” whereby microalgal biomass is used as feedstock is a promising solution to the financial plight of expensive substrates for PHA production (Afreen et al., 2021).

Despite this, to ensure the success of large-scale PHA synthesis using microalgal biomass, a few critical issues have to be taken into consideration such as 1) identifying microalgae strains along with determining optimum growth conditions to ensure maximum growth rates for greater biomass production, 2) employing microalgae cultivation systems together with microalgae harvesting techniques that are both economical and necessitate less management, and 3) selecting the ideal bacteria that work in tandem with the biomass produced by microalgae. Considering this, the following sections will discuss the recent advancement in refining PHA synthesis from microalgal biomass as an industry-scale production that holds economic competitiveness against conventional plastics as well as non-microalgae production systems.

## 2 Microalgae and Bacterial PHA Synthesis

Many studies have been reported on the development of efficient processes for the production of PHA. Microalgae are attractive because of their ability to fix CO_2_ directly to produce biomass. The cultivation of microalgae is especially attractive in tropical countries because of suitable climatic conditions.

### 2.1 Role of Microalgae in the Bacterial Biosynthesis of PHA

The commercial production of PHAs is commonly carried out in large scale by heterotrophic bacteria. While certain natural microalgae species are also able to generate PHAs under stress conditions, the yield is relatively low, ranging from 5 wt% (*Synechocystis* sp. PCC6803) (Sudesh et al., 2002) to a maximum PHA production of 69 wt% (*Nostoc muscorum* Agardh) (Serif et al., 2018) of dry microalgal biomass. Even with genetic engineering, microalgal production of PHA reached a peak of 85 wt% (*Aulosira fertilissima* CCC444) which is lower than bacterial PHA production that can reach over 95 wt% (*Cupriavidus necator*) of cell dry weight (CDW). However, bacterial PHA production is hindered by high price of carbon substrate. This can be solved by utilizing the more economical microalgal biomass for bacterial PHA production.

PHAs are categorized into three clusters depending on their lengths. Short chain length (scl)-PHAs contain three to five carbon atoms with the most commonly synthesized scl-PHA being the homopolymer of poly(3-hydroxybutyrate) [P(3HB)]. In contrast, medium chain length (mcl)-PHAs contain six to 15 carbon atoms. On the other hand, PHA copolymers consist of a combination of different monomer types. For instance, poly(3-hydroxyhexanoate-co-3-hydroxyoctanoate) [P(3HHx-co-3HO)] is a copolymer of mcl monomers while poly(3-hydroxybutyrate-co-3-hydroxyhexanoate) [P(3HB-co-3HHx)] is a combination of scl and mcl monomers. In particular, scl-mcl-PHAs are highly sought for their similar biophysical properties to common plastics such as negligible water solubility with high resistance towards moisture and hydrolytic degradation (Surendran et al., 2020). The most common types of bacterial PHAs produced using microalgal feedstock are the scl copolymer poly(3-hydroxybutyrate-co-3-hyroxyvalerate) [P(3HB-co-3HV)] and poly-(R)-3-hydroxybutyrate (P3HB) (Table 1). With 60% crystallinity, P3HB, is thought to be a suitable substitute to PP. It has a melting temperature of 175°C and transition temperature of 0–9°C (Madadi et al., 2021). On the other hand, P(3HB-co-3HV) is more flexible with a melting temperature between 148 and 168°C and transition temperature of −5.5 to −2.2°C, making it more commercially profitable (Samantaray and Mallick, 2012).

**TABLE 1:** Types of PHA produced according to the bacterial strain and microalgal carbon source.

Algae feedstock	Nutrient used	Bacterial strain	Type of PHA produced	References
Defatted *Chlorella* biomass	Reducing sugars	*Paracoccus* sp. LL1	P(3HB-co-3HV)	Khomlaem et al. (2021)
*Corallina mediterranea*	Reducing sugars	*Halomonas* sp.	P(3HB-co-3HV)	Abd El-Malek et al. (2021)
*Laminaria japonica* biomass	Reducing sugars	*Paracoccus* sp. LL1	P(3HB-co-3HV)	Muhammad et al. (2020)
*Ulva* sp.	Reducing sugars	*Haloferax mediterranei*	P(3HB-co-3HV)	Ghosh et al. (2019)
*Jatropha* biodiesel waste	Reducing sugars	*Halomonas hydrothermalis* MTCC 5445	P(3HB-co-3HV)	Bera et al. (2015)
*Gelidium amansii*	Reducing sugars	*Bacillus megaterium*	P3HB	Alkotaini et al. (2016)
*Gelidium amansii*	Reducing sugars	*Saccharophagus degradans*	P3HB	Sawant et al. (2018)
*Sargassum* sp.	Reducing sugars	*Cupriavidus necator* PTCC 1615	P3HB	Azizi et al. (2017)
Algal biodiesel waste residue	Glycerol	*Halomonas ventosae*	PHB	Dubey and Mishra, (2021)
Algal biodiesel waste residue	Glycerol	*Halomonas daqingensis*	PHB	Dubey and Mishra, (2021)
*Laminaria japonica* biomass	Reducing sugars	*Bacillus megaterium*	PHB	Muhammad et al. (2020)
*Laminaria japonica* biomass	Reducing sugars	*Cupriavidus necator*	PHB	Muhammad et al. (2020)

The PHA synthases of heterotrophic bacteria fall under four classes; PHA synthases of class I, III, and IV polymerize scl monomers, whereas class II polymerizes mcl monomers. Due to this, heterotrophic bacteria can utilize a wide range of substrates such as monosaccharides, starch, glycerol, and fatty acids for PHA production, many of which can be derived from microalgal biomass. PHAs of different composition are produced depending on the carbon substrate supplemented as well as the PHA-producing microorganism (Surendran et al., 2020).

In general, microalgal biomass is rich in various proteins (10–47 wt% of CDW), starch components (10–20 wt% of CDW), and amylopectin (80–90 wt% of CDW), cellulose, and lipids (20–50 wt% of CDW) (Wang et al., 2016; Sun et al., 2018a; Madadi et al., 2021). Table 2 depicts the macromolecule composition of different microalgal strains. Microalgal carbohydrates are the most commonly used carbon sources from microalgal biomass for PHA-synthesizing bacteria. For instance, defatted *Chlorella* biomass was pretreated to yield fermentable sugars which were fed to PHA-synthesizing bacteria to produce P(3HB-co-3HV) (Naduthodi et al., 2019). Furthermore, various microalgal strains contain monosaccharides including glucose, mannose, rhamnose, galactose, arabinose, and xylose. Sucrose has also been extracted from *Desmodesums* and *Scenedesmus* (Smachetti et al., 2020). Many bacteria such as *Azeobacter vinelandii* and *Alcaligenes latus* can synthesize PHA from sucrose (Winnacker, 2019). *A. latus* can also utilize starch to synthesize P3HB (Chen, 2009). Galactose and glucose can be utilized by bacteria such as *Hydrogenophaga pseudoflava* DSM 1034 and *Pseudomonas hydrogenovora* (Koller et al., 2008; Povolo et al., 2013). Recently, crude glycerol from the algal biodiesel industry has been found to be a suitable feedstock for PHA production. PHB was produced by *Halomonas daqingensis* and *Halomonas ventosae* when fed with algal biodiesel waste residue that is rich in glycerol (Dubey and Mishra, 2021).

**TABLE 2:** Commonly employed microalgae for biomass generation and their contents.

Microalgae	Carbohydrate (%)	Lipid	Protein (%)	References
*Anabaena* sp*.*	30.60	26.64%	34.99	Tiwari et al. (2019)
*Arthrospira platensis*	56.56	3.51%	32.90	Rempel et al. (2018)
*Arthrospira* sp*.*	23.90	5.80%	70.30	Feng et al. (2018)
*Botryococcus braunii*	23.39	—	37.00	Ruangsomboon et al. (2017)
*Chlamydomonas reinhardtii*	52.20	22.11%	23.69	Banerjee et al. (2021)
*Chlorella pyrenoidosa*	19.40	11.30%	62.30	Tan et al. (2019)
*Chlorella sorokiniana*	20.20	22.40%	49.50	Gao et al. (2021)
*Chlorella vulgaris*	56.70	8.30%	20.20	Canelli et al. (2020)
*Chromochloris zofingiensis*	13.20	38.40%	13.00	Sun et al. (2020)
*Galdieria sulphuraria*	20.00	3.00%	37.00	Pleissner et al. (2021)
*Nannochloropsis* sp.	18.10	20.70%	48.30	Li et al. (2020a)
*Neochloris oleoabundans*	64.80	15.90%	62.50	Desai et al. (2019)
*Nostoc* sp.	44.91	14.85%	41.33	Silambarasan et al. (2021)
*Porphyridium purpureum*	35.00	1.10%	13.1	Ferreira et al. (2021)
*Phaeodactylum tricornutum*	7.85	9.08%	38.40	Branco-Vieira et al. (2020)
*Scenedesmus obliquus*	33.78	22.27%	41.93	Abomohra et al. (2018)

### 2.2 Choosing the Right Bacterium

To make full use of the microalgal biomass, it is crucial to determine suitable PHA-producing bacteria that can utilize the most of the microalgal nutrients. The general population of PHA-producing microorganisms are able to utilize simple sugars and some are able to consume triglycerides while hydrocarbon utilization for PHA synthesis is rare (Jiang et al., 2016). *C. necator* is one of the commonly used bacterium for industrial PHA synthesis and is deemed as the model organism for PHA metabolism (Reinecke and Steinbuechel, 2009). It stores PHA up to 96 wt% of its CDW when given excess carbon source while being starved of nitrogen or phosphate. The use of genetic manipulation has further increased the commercial potential of *C. necator* in PHA synthesis. The glucose-utilizing mutant, *C. necator* NCIMB 11599, was able to accumulate PHA up to 49 wt% of CDW using the brown algae *Laminaria japonica* biomass as carbon source (Muhammad et al., 2020). Another strain, *C. necator* PTCC 1615, successfully utilized brown seaweed *Sargassum* sp. as feedstock for PHB production (Azizi et al., 2017). *C. necator* KCTC 2649 was able to produce 75.4 wt% of CDW of PHA by using 10% (w/v) of defatted *Chlorella* biomass (Khomlaem et al., 2021). Likewise, *C. necator* TISTR 1335 was fed a combination of *Chlorella* sp. biomass co-digested with sugarcane leaves to produce 60.9 wt% of PHA which contributes to the zero-waste generation concept (Sitthikitpanya et al., 2021).

Halophilic bacteria have gained attention as a candidate for PHA synthesis owing to their unique growth conditions that reduces the chances of contamination. Members of the halophilic Halomonadaceae family are able to amass large quantities of PHAs from different carbon sources (Abd El-Malek et al., 2021). Recently, production of biodiesel from microalgae is gaining worldwide attention as it has been shown to be the only renewable biodiesel source able to meet the global demand for transport fuels. A major by-product of this production is crude glycerol. *H. daqingensis* was found to be able to synthesize PHA by utilizing glycerol-rich algal biodiesel waste residue as the sole carbon source (Dubey and Mishra, 2021). Moreover, *Halomonas pacifica* ASL10 and *Halomonas salifodiane* successfully produced PHAs from the macroalgae *Pterocladia capillaceais* and *Corallina mediterraneais* as well as *Arthrospira* (Abd El-Malek et al., 2021).

The *Paracoccus* species is another potential workhorse for PHA production. In addition to the ability to switch between autotrophic and heterotrophic growth, this Gram-negative species is methylotrophic with denitrifying capabilities and as such is commonly used to treat wastewaters (Kim et al., 2015). *Paracoccus denitrificans* and *Paracoccus pantotrophus* are well studied for their ability to accumulate PHA by utilizing numerous carbon sources, such as glycerol, methanol, *n-*penthanol, and CO_2_ (Kim et al., 2015; Chang et al., 2020). Using *L. japonica* biomass, *Paracoccus* sp. LL1 was able to synthesize PHA as well as carotenoids (Muhammad et al., 2020). Fascinatingly, this same species was able to utilize defatted *Chlorella* biomass to produce 37.4 wt% of CDW of PHA and 6.08 mg·L^−1^ of carotenoids (Khomlaem et al., 2021). These findings highlight the compatibility of using microalgal biomass as feedstock for PHA production in *Paracoccus.*

The wild type bacterium *E. coli* is unable to synthesize PHA. However, transmutating the bacterium with PhaC gene allows for the production of PHA. This principle is applied for *E. coli* XL1-Blue harbouring *phaCAB* from *C. necator* (Spiekermann et al., 1999). By employing the aqueous fraction from an algal wet lipid extraction technique as the medium, PHB production of this particular strain saw an increase of 51% (Sathish et al., 2014). Furthermore, this same recombinant strain was also able to utilize wastewater microalgae to produce the PHB with a maximum accumulation of 31 wt% of the CDW (Rahman et al., 2015).

PHA synthesis is not only limited to Gram-negative bacteria. The Gram-positive *Bacillus* are also known for their PHA production, although not all genus can accumulate PHA in their cells under limiting growth environments. *Bacillus pumilus* (E10) isolated from the wastewaters of University of Santa Cruz do Sul was able to utilize the hydrolysate of *A. platensis* biomass in conjunction with glucose and glycerol to produce PHB. The soil bacterium, *Bacillus megaterium* ALA2, could make use of defatted *Chlorella* biomass and *L. japonica* biomass with PHA production of 29.7 wt% and 32 wt% of CDW, respectively (Muhammad et al., 2020; Khomlaem et al., 2021).

## 3 Microalgae as Feedstock Biofactories

Like most plants, microalgae can grow photoautotrophically but at higher rates which makes them attractive as a source of biomass. In addition, the cellulose in microalgae is more accessible compared to plants. Therefore, much interest and efforts have been directed to the production and use of microalgae biomass as feedstock in various processes.

### 3.1 Wild Type Microalgae

**FIGURE 1:**
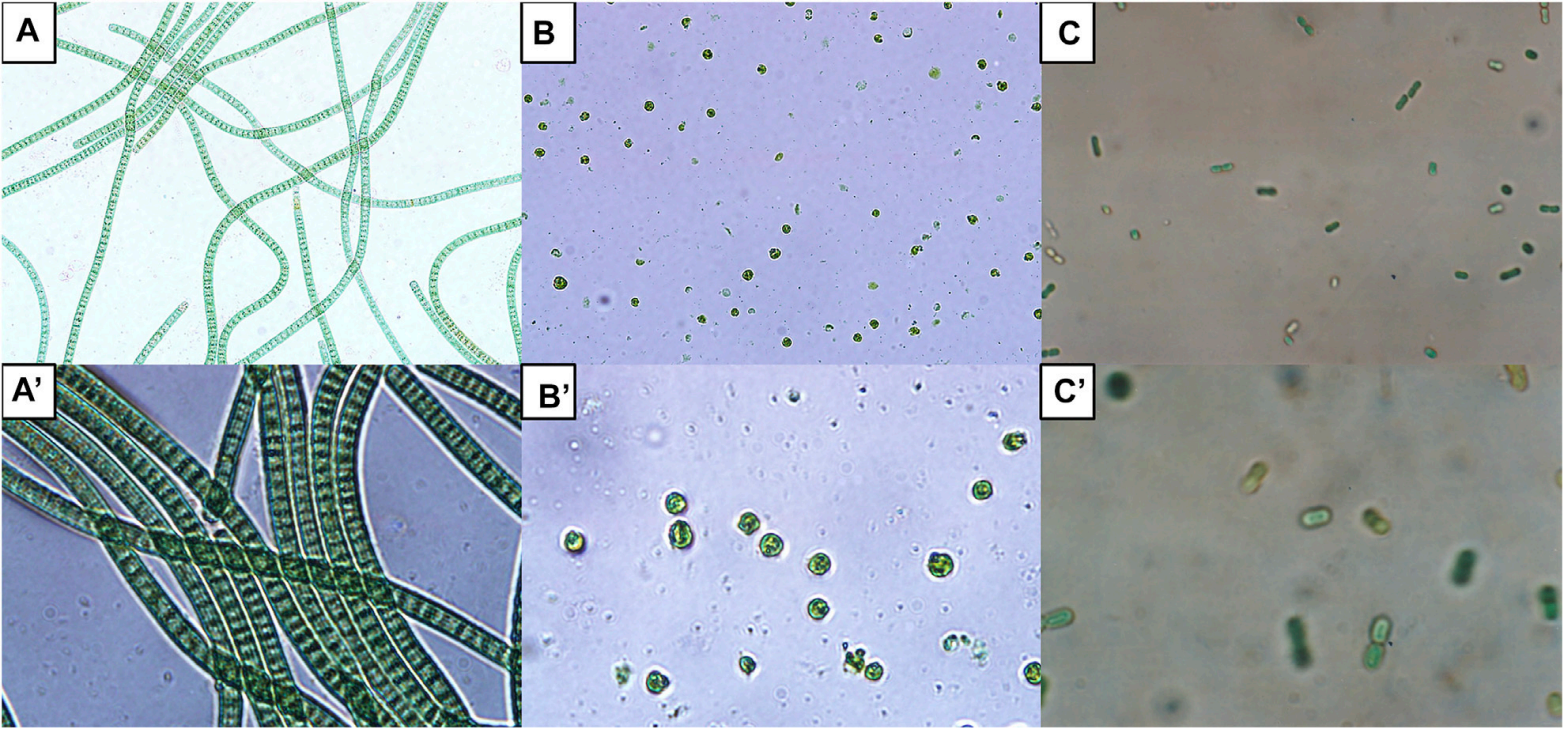
Morphology of some microalgae commonly used for biomass generation viewed under light microscope. *Spirulina (Arthrospira platensis)* UMACC 161 **(A)** total magnification of ×400 and **(A′)** total magnification of ×1000; *Chlamydomonas reinhardtii* **(B)** total magnification of ×400 and **(B′)** total magnification of ×1000; *Synechocystis* sp. strain PCC6803 **(C)** total magnification of ×400 and **(C′)** total magnification of ×1000.

Different microalgae give rise to various types of biomasses. Choosing the right microalgae is crucial in determining the maximum biomass productivity and relative composition of the biomass constituents which then define the end product of the downstream PHA synthesis. Presently, the common wild types that provide high biomass productivity include *Arthrospira*, *Chlorella*, and *Chlamydomonas reinhardtii.* Figure 1 shows the morphologies of *A. platensis*, *C. reinhardtii*, and *Synechocystis* sp. strain PCC6803.

#### 3.1.1 *Arthrospira*

Formerly known as *Spirulina*, *Arthrospira* are filamentous cyanobacteria that thrive in salt lakes but can also be found in freshwaters (Ciferri and Tiboni, 1985). They are generally cultivated as nutritional supplements for their rich protein and vitamin content (Marles et al., 2011). It has been shown that *Arthrospira* can be produced up to 15,000 tons of dry weight annually (Lu et al., 2011). Members of *Arthrospira* can withstand extreme alkaline and saline conditions which makes their cultivation culture free from common contaminants and are thus typically used as microalgae cultures for open ponds cultivation (Feng et al., 2018). Despite its significantly lower lipid content (4–6%), *Arthrospira* biomass tend to accumulate large amounts of carbohydrate under nutrient stress condition (∼up to 70%). In light of this, *Arthrospira* offers a suitable candidate as feedstock for bacterial PHA synthesis (Heasman et al., 2000).

#### 3.1.2 *Chlorella*

*Chlorella* are among the pioneer algae used for commercial applications in open ponds (Guccione et al., 2014). They are spherical single-celled green microalgae. Similar to *Arthrospira*, *Chlorella* biomass has been used as dietary supplements. These microalgae offer a fast biomass growth rate with daily productivity of 25 g·m^−2^ and annual production of more than 2,000 tons (Zhou et al., 2011; Deshmukh et al., 2021). The composition of *Chlorella* is rich in saturated and unsaturated C18 fatty acids which is comparable to vegetable oils and are often used as oil substitutes (Liang et al., 2009).

#### 3.1.3 *Nannochloropsis*

*Nannochloropsis* are a genus of microalgae found in both freshwater and brackish water environments. Members of *Nannochloropsis* have long been used in biopharmaceutical applications for their bioactive compounds such as eicosapentaenoic acid that provide positive health benefits (Rodolfi et al., 2009; Hulatt et al., 2017). Recently, much interest has focused on *Nannochloropsis* as aquaculture feed and biodiesel production for their rapid growth rate, high lipid content, and resistance towards various irradiation conditions (Ma et al., 2014). The annual growth rate of *Nannochloropsis* is approximately 0.16 g·L^−1^∙d^−1^, peaking at 0.37 g·L^−1^∙d^−1^ (Quinn et al., 2012).

#### 3.1.4 *Phaeodactylum tricornutum*

*P. tricornutum* is a marine diatom and the sole species of the genus *Phaeodactylum.* They are unique diatoms as they can survive without silicon and thus lack silicified frustules. It is rich in fucoxanthin, eicosapentaenoic acid, and chrysolaminarin that have extensive beneficial health effects (Zhang et al., 2018). *P. tricornutum* has a biomass composition of 7.85% carbohydrates, 38.40% proteins, and 9.08% lipids as well as a maximum biomass density of 0.6 g·L^−1^ and 1.0 g·L^−1^ when grown in open ponds and photobioreactors, respectively (Branco-Vieira et al., 2020). With a fully sequenced whole genome, *P. tricornutum* is a model photosynthetic representative for non-green algae. For instance, the synthesis of carbohydrate metabolism pathway was discovered using *P. tricornutum* which aids in the understanding of improving carbohydrate accumulation (Kroth et al., 2008).

### 3.2 Bioengineering Enhances Biomass Production

While optimization of microalgae growth factors is known to deliver peak biomass productivities of wild-type microalgae, bioengineering pushes these limits to overcome disadvantages that come with specific microalgae strains. Genetic engineering or genetic modification directly alters an organism’s genes via biotechnology tools. In terms of microalgae, genetic engineering aims to increase biomass productivities by modifying genes associated with photosynthesis, resistance towards extreme conditions and metabolism.

Currently, about 30 microalgae species have fully sequenced genomes. Among them are *Chlorella vulgaris* (Guarnieri et al., 2018), *C. reinhardtii* (Merchant et al., 2007), *Dunaliella salina* (Polle et al., 2017), *Synechocystis* sp. strain PCC6803 (Kaneko and Tabata, 1997), and *Scenedesmus obliquus* (Starkenburg et al., 2017). The pioneer of microalgae DNA modification was *C. reinhardtii* by Rochaix and van Dillewijn over 30 years ago (Rochaix and Van Dillewijn, 1982). Since then, more tools were developed to enhance the yield of *C. reinhardtii* but few of these approaches are viable for other microalgae.

Bioengineering methods widely employed for gene sequence modification in microalgae include Clustered Regularly Interspaced Short Palindromic Repeats—CRISPR associated with the protein 9 (CRISPR–Cas9) (Shin et al., 2016), zinc-finger nuclease (ZFN) (Sizova et al., 2013), Transcription Activator-Like Effector Nucleases (TALENs) (Daboussi et al., 2014) whereas RNA interference techniques such as microRNAs (RNAi) and short interfering RNAs (siRNA) are used to activate or repress expression of certain genes (Kim et al., 2015).

Compared to TALENs and ZFN, the CRISPR approach had higher applicability, allowing modulation of multiple gene expressions. However, the use of CRISPR editing for microalgae is obstructed by the toxicity of the Cas9 nuclease which results in a 10% mutation rate (Jiang et al., 2014). To overcome this, Cas9 protein-gRNA ribonucleoproteins (RNPs) provided an alternative to the lethal Cas9 (Jiang et al., 2014). Cas9 RNPs were successfully delivered into *C. reinhardtii* with an improved mutation rate of almost 100-fold compared to the general Cas9 approach (Shin et al., 2016). Following this success, RNPs were adopted for gene editing of other microalgae including *Nannochloropsis oceanica* IMET1 (Naduthodi et al., 2019), *P. tricornutum* (Serif et al., 2018), and *Tetraselmis* sp. (Chang et al., 2020).

Regardless of the recent developments in genetic engineering techniques for microalgae, bioengineering microalgae is still at its infancy stage with only a few fully sequenced genome species in addition to their complicated anatomy and physiology that hinder most genetic engineering tools. With the advancement and evolving CRISPR technology and the combination of different genetic tools, it is expected that genetic engineering will reach a breakthrough for more efficient developments of commercially-sustainable genetically engineered microalgae.

## 4 Optimization of Microalgal Growth Conditions

Microalgae development is affected by both biotic and abiotic parameters. Biotic parameters comprise of stresses from pathogens including viruses, detrimental bacteria, fungi as well as other microalgae. Contrariwise, abiotic factors cover parameters, such as light quantity and quality, pH, salinity, temperature, carbon dioxide, dissolved oxygen and availability of nutrients. While regulating biotic parameters is essential for healthy growth, different algae species require specific abiotic factors. Therefore, optimization of these growth requirements is required to improve biomass output. Paliwal et al. has documented how abiotic stress is used for maximizing lipid and fatty acid production (Paliwal et al., 2017).

### 4.1 Light

Photosynthetic organisms utilize light as the main source of energy. The most crucial aspect in microalgae growth is arguably light in the form of light limitation, saturation, and inhibition (Gatamaneni et al., 2018). The photosynthesis process necessitates both dark and light phases. In the presence of light, the light energy is absorbed by microalgae and assimilated into adenosine triphosphate (ATP) which is utilized for biomass synthesis throughout the dark cycle. As such, the dispensation of light to the cultivation system requires optimization in terms of the system’s geometric design and orientation (Fernández et al., 2001; Tredici et al., 2015). In light limiting environments, microalgae growth is proportional to the increase of light intensity. On the other hand, light saturation conditions diminish photosynthesis as the absorption of photons surpasses electron turnover (Chang et al., 2017). Further light over-exposure leads to permanent impairment to the photosynthetic system in a phenomenon termed photo-inhibition (Tredici and Zittelli, 1998). Most microalgae have a saturated photosynthesis rate at 100–500 μE∙m^−2^∙s^−1^ and any excess light exposure will cause the microalgae to be photo-inhibited (Vejrazka et al., 2012).

Artificial or natural (solar) light sources can be utilized in cultivation systems. While solar energy is the most economically and readily available source, artificial light is preferred in high value-added cultures for the accurate regulation of photoperiod and control of the light spectrum (Schulze et al., 2014). Among the many artificial lights available such as halogen lamps and fluorescent lights, light-emitting diodes (LEDs) allows for the best modulation of light with different wavelengths (Schulze et al., 2016). The photosynthetically active radiation (PAR) that most microalgae thrive under is at the wavelength range of 380–750 nm (white light), wavelength range of 420–470 nm (blue light) and wavelength range of approximately 660 nm (red light) (Fu et al., 2013; Schulze et al., 2014). Red to far-red lights result in increased microalgae growth rate with smaller cells and reduced nutrient consumption. Blue light modifies gene expression and specific metabolic pathways resulting in an increased nutrient intake with larger cells but slower growths (Schulze et al., 2016).

Microalgae will light acclimate throughout the production phase in batch cultures; being high light (HL) acclimated consecutively after introduction with new cultures while being low light (LL) acclimated by the end of the batch cycle at high cell density (Grobbelaar et al., 1996). As a result, mega-scale microalgal cultivation systems can retain biomass at low light by maintaining biomass at high concentrations. Alternatively, small-scale microalgal cultivation systems can also employ the microalgal photo-acclimated state to attain exponentially high produce. Using a multi-compartment photobioreactor, the first layer of microalgae facing the light source were HL acclimated and consecutive layers of microalgae became gradually more LL acclimated. This continuous flow photobioreactor design takes advantage of microalgae that are acclimated simultaneously at different light conditions with productivity rates of almost 40% more than the conventional single-layered perpendicular plate reactor (Grobbelaar and Kurano, 2003).

Recent years have also seen an increase in studies on other novel strategies to boost light utilization by microalgae and thereby increasing productivities. Table 3 shows a number of these approaches. In raceway ponds, addition of light filters increased the productivity of *Chlorella* (32.6% increase in biomass productivity) and *D. salina* (68% cell weight increment) (Cheng et al., 2015b; Nwoba et al., 2021). When grown in photobioreactors with light/dark regulation, *Chlorella* experienced a 21.6% increase in biomass productivity. The use of light-splitting/light-harvesting additives also aids in increase in biomass productivities. Silicon dioxide nanoparticles increased biomass productivity of *Scenedesmus* by 22.3% while calcium carbonate crystals raised biomass activity of *Neochloris oleoabundans* by 31.5% (Hong et al., 2019; Ren et al., 2020).

**TABLE 3:** Recent strategies to enhance light utilization and increase productivities in microalgae.

Strategy	Microalgal	Result	References
Optimization of lights red: green: blue at a ratio of 80:10:10	*Chlamydomonas reinhardtii*	Biomass productivity: 0.252 g·L^−1^∙d^−1^	Baer et al. (2016)
Novel photobioreactor design that regulates light/dark cycle	*Chlorella pyrenoidosa*	Biomass productivity increment: 21.6 ± 2.1%	Liao et al. (2014)
Flashing light effect with up-down chute baffles in raceway ponds	*Chlorella* sp.	Biomass productivity increment: 32.6%	Cheng et al. (2015b)
Organic dye as wavelength converters	*Chlorella vulgaris*	Lipid productivity increment: 30%	Seo et al. (2015)
Optimization using LED warm light	*Chlorella vulgaris*	Photosynthetic rate: 0.275	Khalili et al. (2015)
Embedding hollow light guides to a flat-plate photobioreactor	*Chlorella vulgaris*	Photosynthetic efficiency increment: 12.52%	Sun et al. (2016)
Growth-phase based light-feeding	*Chlorella vulgaris*	Lipid productivity increment: 52.38%	Sun et al. (2018b)
Light-harvesting gold nanoparticles	*Chlorella zofingiensis*	Carotenoids production increment: 42.7%	Li et al. (2020b)
Monochromatic light filters in raceway ponds	*Dunaliella salina*	Cell volume increment: 200%, cell weight increment: 68%, chlorophyll a enhancement - 35%, protein increment: 51%	Nwoba et al. (2021)
Optimization of red light	*Galdieria sulphuraria*	Biomass productivity: 0.252 g·L^−1^∙d^−1^	Baer et al. (2016)
Usage of light-splitting CaCO_3_ ^a^ crystal	*Neochloris oleoabundans*	Biomass productivity increment: 31.5%, lipid increment: 18.4%	Hong et al. (2019)
Optimization of lights red: green: blue at a ratio of 40:40:20	*Porphyridium purpureum*	Phycocyanin productivity: 0.304 g·L^−1^∙d^−1^	Baer et al. (2016)
Night illumination using monochromatic light-emitting diodes	*Scenedesmus obliquus*	Biomass productivity: 0.198 ± 0.005 g·L^−1^∙d^−1^	Abomohra et al. (2019)
Optimization using white LED	*Scenedesmus obliquus* FSP-3	Lutein productivity of 0.004 g·L^−1^∙d^−1^	Ho et al. (2014)
Addition of SiC^a^ nanoparticles under xenon lamp illumination	*Scenedesmus* sp.	Biomass productivity increment: 22.3%, lipid productivity increment: 42.2%	Ren et al. (2020)
Light intensity: 420 μmol m^−2^ s^−1^	*Scenedesmus obliquus* CNW-N	Maximum carbohydrate productivity: 0.322 g·L^−1^∙d^−1^	
Usage of light filters	*Tetraselmis* sp. KCTC12236BP	Biomass productivity increment: 53%	Kim et al. (2017)
White fluorescence tubes at 150 μE m^−2^ s^−1^	*Tetraselmis subcordiformis*	Starch productivity: 0.011 g·L^−1^∙d^−1^	Zheng et al. (2011)
Response surface methodology and central composite face–centered design	*Ettlia* sp*.*	Biomass productivity: 28.0 ± 1.5 g·L^−1^∙d^−1^	Kim et al. (2018)

a CaCO_3_ denotes calcium carbonate, SiC denotes silicon carbide.

Rise in light intensity yielded an increase in carbohydrate generation with light intensity in the range of 30–400 μmol·m^−2^·s^−1^ boosts the accumulation of carbohydrates. For instance, *S. obliquus* CNW-N proved that there was a positive association between biomass/carbohydrate productivities and light intensity prior to photoinhibition; high light intensity of 420 μmol·m^−2^·s^−1^ led to peak carbohydrate productivity of 0.322 g·L^−1^∙d^−1^ which was higher than that of *Tetraselmis subcordiformis* (0.256 g·L^−1^∙d^−1^) (Zheng et al., 2011; Ho et al., 2012).

### 4.2 Temperature

Temperature regulates biochemical processes of microalgae, particularly the gross photosynthetic rate through cellular division, which in turn affects biomass production. Culturing microalgae at lower temperatures than optimum will affect photosynthesis as carbon assimilation activity is reduced while overheating degrades photosynthetic proteins which lowers photosynthetic rates and thereby shrinks microalgae cells (Anjos et al., 2013). Optimal temperatures for most microalgae species range between 20 and 35°C (Xu et al., 2010; Cho et al., 2011) but certain thermophilic species such as *Anacystis nidulans* can tolerate up to 40°C (Caïa et al., 2018).

Microalgae typically absorb radiated heat from the light source. Additionally, microalgae growth is highly exothermic with over 95% of light absorbed converted into heat (Cheng et al., 2015a). Small-scale microalgae cultivation systems may not require temperature control as heat is released to the environment through convection when the surrounding setting is cold enough. However, mega-scale outdoor cultivation of microalgae is constantly exposed to solar radiation which directly heats cultures (Chiang et al., 2011). In essence, closed systems are inclined to overheat while open systems experience high water evaporation rates under intense irradiance (Gonzalez-Camejo et al., 2020). Since higher temperatures are more lethal to microalgae than lower temperatures, culture cooling is employed particularly with closed photobioreactors. Nevertheless, lower temperatures (<10°C) result in reduced biomass productivities (Thompson, 1996). Furthermore, temperature highly affects enzymes involved in starch production such as starch synthase and sucrose synthase. When temperature was increased from 5 to 20°C, carbohydrate content in *C. vulgaris* SO-26 plummeted from 70 to 50% (Madadi et al., 2021). As such, depending on the surrounding climate, heating during winter and cooling during summers are advantageous to microalgae culturing.

Much effort was made to identify the best method to prevent overheating of cultures in large-scale settings. The typical approach is using water sprays to sprinkle the surface of the photobioreactors with heated or cooled liquid but this is only suitable for sites of low air humidity (Cheng et al., 2019b). Heat exchangers are also widely employed to dissipate excess heat to large water bodies. Both open and closed cultivation systems in temperate regions can be housed in greenhouses (Gerardi, 2015; Cheng et al., 2019a).

Recent technologies have allowed for in-depth studies on the effects of temperature on microalgae culture conditions; be it in a controlled laboratory setting, outdoor systems, photobioreactor simulations, and theoretical models. For instance, cultivation of *Arthrospira platensis* in winter saw an increase of phycocyanin productivity when the microalgal was grown in a thermally-insulated photobioreactor complimented by photovoltaic plate incorporation (Gensemer et al., 1993). Another study reported increased photosynthetic conversion efficiency of over 7% from cultures with *Scenedesmus* and *Chlorella* species when grown in a photobioreactor fixed with a double-wall hose structure combined with temperature control in a closed system (Anderson and Morel, 1978). Furthermore, thermal modeling aids in simulating and optimizing various reactor designs. One such model delved into the effect of different flat-plate reactor designs which identified accurate predictions of monthly energy consumption needed to regulate the temperature of microalgae cultures (Rotatore and Colman, 1991).

### 4.3 Carbon Dioxide

Microalgal biomass production is unique as microalgae are capable of fixing carbon dioxide (CO_2_). As mentioned, the CO_2_ fixing efficiency of microalgae is higher than terrestrial plants. Consequently, carbon dioxide is one of the limiting reactants in photosynthesis. Concentrations of CO_2_ needed for peak photosynthetic efficiency is 1–5 vol% (Solimeno et al., 2015). Given this, the available CO_2_ in the air of only 0.04 vol% is insufficient for high productivity (Xu et al., 2010). CO_2_ can be supplied as atmospheric air, commercially purified CO_2_, raw flue gas or via supplementation of salts, for instance, bicarbonates (Cho et al., 2011).

The demand for CO_2_ for microalgae growth is at its highest during the day when photosynthetic activity is active, while there is zero demand at night. CO_2_ delivery approaches must manage these demands that fluctuate seasonally and diurnally. Open raceway ponds and closed systems often use spargers or diffusers to deliver CO_2_ (Chiang et al., 2011; Anjos et al., 2013). The sparger injects CO_2_ in gas bubbles at the bottom of the pond while a paddlewheel circulates CO_2_ throughout the microalgae culture (Cheng et al., 2015a). By studying CO_2_ transfer rates in an open algal pond, it was found that decelerating the paddle wheel rotation speed to 13 rpm decreases CO_2_ and losses up to 61% (Caïa et al., 2018). Additionally, to achieve the maximum CO_2_ utilization efficiency, CO_2_ must be extracted from gas bubbles before the bubbles escape to the pond surface. Sumps were introduced at the point of gas injection to lengthen the retention time of bubbles (Thompson, 1996). Another strategy was to minimize the bubble size. Microbubbles of diameters not larger than 100 μm have a higher surface-to-volume ratio and rise slowly to the surface of culture, allowing more CO_2_ to disperse throughout the medium (Gonzalez-Camejo et al., 2020). Novel photobioreactors designs have considered these factors when integrating technologies to increase CO_2_ utilization efficiency. Some examples include the jet-aerated tangential swirling-flow plate photobioreactor (Cheng et al., 2019a) that condenses bubble diameter and CO_2_ microbubbles dissolver (CMD) (Cheng et al., 2019b) that facilitates dissolved CO_2_ in photobioreactors.

### 4.4 Hydrogen Potential (pH)

Hydrogen potential (pH) is known to affect not just the microalgae but also influences the mineral and carbon dioxide solubility of the media. Most microalgae tolerate a pH range of 6–10 (Yang et al., 2011). At extremely acidic conditions, the absorption rate of nutrients and trace metals is altered which might cause metal toxicity (Anderson and Morel, 1978; Gensemer et al., 1993). On the other hand, extremely alkaline conditions cause enzyme degradation as well as lowering microalgae affinity towards free CO_2_ (Rotatore and Colman, 1991). During microalgal photosynthesis at optimal pH, available bicarbonate in the medium is transformed into CO_2_ which releases hydroxyl ions. These excess hydroxyl ions increase the pH of the medium (Gerardi, 2015). Carbon at alkaline conditions is present in the form of carbonates and is not favored by microalgae (Solimeno et al., 2015). Supplementation of CO_2_ acidifies the microalgal medium by altering carbonate balance (Galloway and Krauss, 1961). Consequently, controlled injection of CO_2_ is required to maintain optimal pH levels of the medium. The employment of sensors to monitor pH levels has also benefitted microalgae cultivation as seen in a model that observed *C. reinhardtii* cells (Ifrim et al., 2014). Such pH monitors can also aid in determining photosynthetic productivity (Gonzalez-Camejo et al., 2020).

### 4.5 Nutrients

Understanding the key nutrients for microalgae growth would aid in maximizing biomass productivity as well as the production of favored synthesis. Microalgae demonstrate significant fluctuations in biochemical compositions when cultured under different limiting nutrients. Nutrient requirements of microalgae are calculated based on the formula of CO_0.48_H_1.83_N_0.11_P_0.01_ (Ifrim et al., 2014). The three non-mineral nutrients essential for photosynthesis are carbon, oxygen, and hydrogen. Carbon is required in bulk as it is a major component of all organic substances including proteins, carbohydrates, lipids and even nucleic acids (Ifrim et al., 2014). Autotrophic microalgae entail inorganic carbon sources in the forms of CO_2_, carbonate, and bicarbonate while heterotrophic microalgae can use acetate or glucose (Ifrim et al., 2014).

Nitrogen is the second most element in the microalgal biomass which makes up for 7–20% of the dry cell weight (Ifrim et al., 2014). It is the building block of all structural and functional proteins. Nitrogen-depleted microalgae are inclined to carbohydrate synthesis (Ifrim et al., 2014). By limiting NaNO_3_, NaH_2_PO_4_, metals, and vitamins on *Tetraselmis* sp., starch content peaked at 42% of CDW (Dammak et al., 2017). Similarly, starch content of *C. vulgaris* and *Chlorella zofingiensis* increased over 40% and 66%, respectively when starved of nitrogen (Dragone et al., 2011). Nitrogen limitation was also shown to be the most superior inducer of carbohydrate accumulation compared to limiting sulphur or phosphorus (Brányiková et al., 2011; Yuan et al., 2018). *Parachlorella kessleri* that were starved off of nitrogen, phosphorus or sulphur experienced a spike in carbohydrate content for the first few days before dropping. This was then followed by lipid accumulation. The initial reaction might be due to carbohydrate synthesis as energy reserves in response to the initial stress before the same energy is utilized for lipid synthesis if starvation was prolonged (Shaikh et al., 2019).

Phosphorus is a core macronutrient involved in ATP biosynthesis, nucleic acid formation, growth and cellular maintenance. Starvation of inorganic phosphate in *Chlorella* sp. FC2IITG exhibited high carbohydrate content up to 47.35 wt% of CDW (Muthuraj et al., 2014). Deprivation of phosphate on *Leptolyngbya limnetica* and *Oscillatoria obscura* resulted in increased carbohydrate levels of 44.5 wt% and 40.4 wt% of CDW, respectively which were 45% more than carbohydrate levels under nitrogen limitation (Kushwaha et al., 2018).

Additionally, trace elements such as sulphur, copper, and manganese play significant roles in microalgae growth. Sulphur starvation stops cellular metabolism while amassing compounds such as carbohydrates. *C. vulgaris* was reported to amass 60% of carbohydrates when sulphur was limited (Brányiková et al., 2011). Starving of manganese and potassium also instigated an increase in carbohydrate content in *C. reinhardtii* (Markou et al., 2012)*.* Calcium and magnesium limitation also increased carbohydrate concentration of *Chlorella sorokiniana* by 50% without compromising biomass productivity (Hanifzadeh et al., 2018). Conversely, exposure to high copper concentration induced carbohydrate production in diatoms *Cylindrotheca fusiformis* and *Gymnodimium* sp. (Pistocchi et al., 2000). Likewise, high carbohydrate accumulation was reported when iron was in excess in tandem with nitrogen limitation and high light illumination (Yeesang and Cheirsilp, 2011).

### 4.6 Chemical Modulators

In addition to moderating microalgal growth parameters, an alternative technique in refining biomass production is the provision of chemicals. Chemical modulators do not compromise microalgal growth that is observed in nutrient deficiency nor does it necessitate specific data on molecular targets that is required in the genetic engineering method. Instead, chemical modulators are naturally occurring molecules in microalgae that rely on phenotypic screening to target the cellular functions, growth, and metabolism of the microalgae. For instance, a large scale phenotypic screening of 42 chemicals on their functions in microalgal lipid metabolism identified 12 modulators that enhanced over 100% of intracellular lipid levels in addition to successfully up-scaling the use of two chemicals, propyl gallate and butylated hydroxyanisole (Franz et al., 2013). Similarly, 10 chemical modulators were screened on *Scenedesmus dimorphus* UTEX1237 and 6 were found to enhance carbohydrate productivity: butylated hydroxyanisole, forskolin, acetylcholine, brefeldin, propyl gallate, and jasmonic acid (Paliwal and Jutur, 2021). In the same study, butylated hydroxyanisole, forskolin, acetylcholine, brefeldin and propyl gallate are economically feasible. Additionally, naphthoxyacetic acid or jasmonic acid enhanced lipid accumulation of *Schizochytrium* sp. S31 by 11.16% and 12.71%, respectively (Wang et al., 2018). As chemical modulators either act directly on a target enzyme or functions as signal molecules, a novel approach termed “chemical modulator based adaptive laboratory evolution” was developed for *Crypthecodinium cohnii* (Diao et al., 2019). By using the chemical sesamol, this study found that quenching of reactive oxygen species enhanced central carbohydrate and energy metabolism. Conversely, the application of γ-Aminobutyric acid (GABA) along with nitrogen starvation was found to improve starch content of *Tetraselmis subcordiformis* by 23.4% and starch yield by 28.6% presumably by reducing reactive oxygen species (Ran et al., 2020). Considering their low-cost, chemical modulators are appropriate treatments for sustainable microalgal biorefinery.

## 5 Microalgal Biomass Manufacturing Technology

### 5.1 Mass Cultivation of Microalgal Biomass

There are generally two classes of microalgae cultivation systems available: open raceway pond (RWP) and closed photobioreactor (PBR). Closed systems allow for precise control of growth parameters as well as minimal risk of biological and non-biological contamination. Alternatively, open systems make use of simple designs, natural solar illumination, and lower operating costs. Table 4 depicts a summarized comparison between the available microalgae cultivation systems.

**TABLE 4:** Comparison between microalgae cultivation systems.

	Open RWP	Tubular PBR	Flat-plate PBR	Column PBR
Design	1. Depth < 20 cm	Transparent tubes organized in vertical, inclined, helix or horizontal positions	Transparent rectangular-shaped compartments with a depth of 1–5 cm and plate thickness of 16 mm	Clear cylindrical tubing fitted with a gas sparger
	2. Assembled with a paddle wheel to circulate microalgae in a series of continuous loops			
Pros	1. Most energy-efficient	1. Most cost effective PBR	1. High total surface area for efficient light illumination	1. Highly efficient CO_2_ usage and release of O_2_
	2. Easy maintenance	2. Large illumination surface area	2. Low O_2_ accumulation	2. Low capital cost
	3. Low energy inputs	3. Short light path, thus high-density cultures are achieved	3. Ease of sterility	3. Compact
			4. Suitable for outdoor cultures	4. Ease of sterility
				5. High mass transfer
Cons	1. Excessive water loss	1. Large area of land required	1. Poor aeration	1. Low light utilization
	2. Large area of land required	2. Low CO_2_ dissolution	2. Short penetration depths	2. High cost
	3. Low CO_2_ utilization efficiency	3. Limited temperature control	3. Lower yields	3. Intricate set-up
	4. Low light penetrance	4. Poor axial mass transfer	4. Easy fouling of channels	
	5. Susceptible to contamination	5. Easy fouling of channels	5. Difficult to scale up 6. Limited temperature control	
Biomass productivity (g·L^−1^∙d^−1^)	*Nannochloropsis* sp.: 0.25 (Barat et al., 2017)	*Nannochloropsis* sp: 3.03 (Adamczyk et al., 2016)	Chlorella: 0.419 (Do et al., 2022)	*Nannochloropsis* sp: 0.05014 (Yustinadiar et al., 2020)
	*Chlorella*: 0.056 (Chi et al., 2022)	*Chlorella*: 1.251 (Chen et al., 2019)	*Ascochloris* sp.: 0.292 (Kumar et al., 2019a)	*Chlorella*: 0.593 (Nair and Chakraborty, 2020)
	*Ascochloris* sp.: 0.23 (Kumar et al., 2020)	*Arthrospira* platensis: 0.49 (Shabani, 2016)	*Arthrospira*: 0.30 (Song et al., 2021)	*Ascochloris* sp.: 0.284 (Kumar et al., 2019a)
	*Arthrospira* sp.: 0.151 (Olguín et al., 2003)	*Haematococcus pluvialis*:0.55 (López et al., 2006)	*Synechocystis aquatilis*: 3.12 (Zhang et al., 2002)	*Haematococcus pluvialis*: 0.12 (López et al., 2006)
	*Graesiella* sp.: 0.40 (Wen et al., 2016)	*Acutodesmus obliquus*: 0.15 (Sandmann et al., 2021)		*Stichococcus bacillaris*: 0.14 (Olivieri et al., 2013)
	*Scenedesmus rubescens*: 0.020 (Lin and Lin, 2011)	*Scenedesmus obliquus*: 0.44 (Gouveia et al., 2016)		
		*Consortium C*: 0.90 (Gouveia et al., 2016)		

#### 5.1.1 Open Raceway Pond (RWP)

The RWP is the most widely employed open system design. In addition to being more cost-effective than closed PBR, the system has a simplistic operation, requires low energy and is easily scalable (Costa and De Morais, 2014). RWPs are generally assembled with a paddle wheel to circulate microalgae in a series of continuous loops wherein nutrients and fresh microalgal broth are added to the front of the wheel while harvesting occurs behind the wheel (Mata et al., 2010). This design is considered to be the most energy-efficient pond cultivation design as it only requires a single paddle wheel for agitation of a 5-hectare pond (Rogers et al., 2014). The wheel gently mixes the pond culture with high mixing efficiency which minimizes injury to the flocculated microalgae (Morris et al., 1974). However, RWPs suffer from excessive water loss to the environment which affects the CO_2_ utilization efficiency (Tan et al., 2018). There is also relatively low light penetrance throughout the microalgae cultures which creates a “dark zone” at the bottom of the pond (Hadiyanto et al., 2013). Furthermore, the nature of RWPs that is subject to environmental conditions results in inconsistent microalgae growth rates and a high risk in contamination (Chisti, 2007).

Current RWP designs are excavated at a shallow depth of not more than 20 cm for efficient light capture in addition to reducing hydraulic power consumption of the paddle wheel (De La Obra et al., 2017). However, this depth expedites a larger surface area to incorporate large culture volumes, which increases evaporation. While evaporation aids in preventing overheating of the cultivation medium, it is essential for the cultivation water to be refilled regularly. Transparent light-scattering columns (LSC) can mitigate both of these issues. Vertically immersed LSCs not only enhanced brightness throughout the depth of the pond but also decreased up to 13.6% evaporation loss by decreasing the surface area between the air-liquid layer (Sirikulrat et al., 2021).

Open ponds are exposed to the environment and are thus vulnerable to contamination. To combat this, microalgae strains that require specific environments are preferred such as *A. platensis* that thrive in extreme alkaline states or *D. salina* that can tolerate high salinity (Cheng et al., 2019b). Organic pollutants and unwanted microbes can also be removed via solar photo-Fenton by employing iron and hydrogen peroxide to irradiate water (Polo-López and Pérez, 2021).

Dead zones or stagnant zones occur due to imperfect mixing of the culture, leading to sedimentation and anaerobic conditions (Hadiyanto et al., 2013). Such an environment promotes the growth of unwanted anaerobic microbes which results in a drastic drop in biomass production (Becker, 1994). The liquid velocity needed to minimize dead zones must be at least 0.1 m s^−1^ (Weissman et al., 1988). The application of flow deflectors and wing baffles at each bend of the pond promotes higher consistency of the velocity flow throughout the pond (Hadiyanto et al., 2013).

#### 5.1.2 Photobioreactors (PBR)

PBRs are enclosed vessels supplied with artificial light (Kilham et al., 1997). A PBR is made up of four main phases; the solid phase of microalgal cells, the liquid phase with culture medium, the gaseous phase consisting of CO_2_ and O_2_, and a light-radiation field (Wu et al., 2013). The closed systems were originally introduced to solve complications from the open system ponds (Chisti, 2007). PBRs are compact and space-efficient which does not require large land masses (Wu et al., 2013). The system also allows for a highly controlled growth environment (Wu et al., 2013). This makes the microalgal cultures less prone to contamination as well as allows for optimized growth conditions which results in higher biomass production. Designs of efficient PBRs require optimization of the mixing state for improved effective CO_2_ mass transfer, strengthening the flashlight effect whereby cultures experience the transition between light and dark regions, maintenance of a good nutrient distribution, and prevention of culture sedimentation (Ugwu et al., 2008). Regardless, utilization of PBRs is restricted by its limited scalability and immense capital costs and high capital (Ciferri and Tiboni, 1985).

Tubular PBRs are the most commonly used closed system design. They are constructed as transparent long tubes known as solar collectors which are organized in vertical, inclined, helix, or horizontal positions for efficient light capture (Tarantino, 2003). Microalgal culture is circulated in a constant loop from the reservoir to the solar collector via a mechanical pump or an airlift structure (Chisti, 2007). The recycling of microalgal culture allows the exchange of CO_2_ and O_2_ while sustaining the mixing process (Marles et al., 2011). To avoid photooxidation, the culture is also continuously directed to a degassing column to eliminate the collected O_2_ while cooling water is propelled into the column as a temperature regulator (Chisti, 2007). In efforts to further optimize the consumption of dissolved O_2_, a novel electrochemical tubular PBR that employs anion-exchange membrane alkaline fuel cells coupled with Pt40Ru20 as the cathode catalyst was able to reduce dissolved O_2_ content from 20.0 to 10.72 mg·L^−1^ in 45 min (Feng et al., 2018). Another design addresses the drawback of low CO_2_ dissolution which leads to deficient carbon sources and thereby inhibiting microalgal growth. Here, the use of ZIF8-SE medium comprising of zeolitic imidazolate framework-8 (ZIF-8) nanoparticles increased CO_2_ mass transfer resulting in a 25.5% increase in biomass yield (Xu et al., 2021). Similarly, an innovative spiral-ascending CO_2_ dissolver was established to enhance the CO_2_ mass transfer and extend gas−liquid contact time. This tubular PBR design markedly improved biomass accumulation by 40.8% (Xu et al., 2020).

Flat-plate PBRs are transparent rectangular-shaped compartments with a depth of 1–5 cm that are positioned horizontally or vertically (Lu et al., 2011). The plate is thin with a thickness of 16 mm to allow optimum radiance penetration (Deshmukh et al., 2021). Mixing of microalgal culture is achieved by an airlift system (Zhou et al., 2011). Flat-plate PBRs boost a high total surface area for efficient light illumination as well as low O_2_ accumulation. However, poor aeration and short penetration depths result in lower yields compared to conventional tubular PBRs. To resolve this, a novel flat-plate PBR utilized double paddlewheels to promote mass transfer and increased horizontal fluid velocity between light/dark zones. This addition of paddlewheels augmented the microalgal growth rate by 121.1% (Cheng et al., 2018). Besides this, a novel jet-aerated tangential swirling-flow plate PBR design was shown to reduce average bubble diameter by 80.2% and enhance mass transfer coefficient by 4.6 times (Cheng et al., 2019a).

The column PBR is designed as clear cylindrical tubing fitted with a gas sparger that mixes and agitates the microalgal culture by propelling in air bubbles (Mohan et al., 2019). Typical column PBR designs include only the sparger and no other internal structures. This system allows for strong gas-liquid mass transfer, highly efficient CO_2_ usage and release of O_2_, inexpensive capital cost, and low shear forces (Singh and Sharma, 2012; Moreno-Garcia et al., 2017). Unfortunately, the cylindrical structure has limited efficiency of light utilization and therefore requires high energy to achieve adequate lighting (Huang et al., 2017). Due to this, the tubing cylinder diameter is limited to 0.2 m to while the maximum height is 4 m for structural support (Wang et al., 2012; Moreno-Garcia et al., 2017). By attaching an internal light column, a novel column PBR design enhanced light intensity in the column and increased biomass production by 82.4% (Li et al., 2018). Another design employed a serial lantern-shaped draft tube that improves flashing light in column PBR (Ye et al., 2018). This yielded a 74% increment in biomass production.

### 5.2 Microalgal Biomass Harvesting Techniques

Harvesting is the process of separating microalgae from their growth media. This generally involves the elimination of water from the microalgal medium which thereby concentrates the biomass. Due to the minute sizes of microalgae (diameters of 3–30 m), their cell density is similar to water which poses a challenge in the recovery process (Moreno-Garrido, 2008). Harvesting cost accounts for at least 20% of total microalgal biomass cost and can even reach up to 90% of total costs for open RWPs (Rodolfi et al., 2009; Hulatt et al., 2017). As such, the selection of a suitable harvesting method must consider the overall energy consumption and properties of the chosen microalgal, cell size and density, final product specifications and reusability of the culture medium (Quinn et al., 2012; Ma et al., 2014).

#### 5.2.1 Centrifugation

Centrifugation separates microalgal cells from the media depending on the particle size and density, microalgal species and type of centrifuge used (Heasman et al., 2000; Soomro et al., 2016). This technique offers many advantages including high cell separating efficiency (over 90%), chemical-free biomass and applies to all microalgae.

Disc stack centrifuges are the most utilized industrial centrifuge for commercially valuable algal products (Hulatt et al., 2017). With high centrifugation forces of 4,000 to 14,000 times gravitational force, this method has a low separation time and is ideal for extracting particles of sizes between 3 and 30 µm and low concentrations of 0.02–0.05% of microalgae cultures with a maximum of 15% solids (Milledge and Heaven, 2013). Moreover, a disc stack centrifuge effectively separated solid/liquid, liquid/liquid and liquid/liquid/solid by applying high centrifugal forces in a single continuous course (Sharples and Doman Road, 1991). As a result, these centrifuges require higher energy consumption than other centrifuges. Calculations from using a Westfalia HSB400 disc-bowl centrifuge observed that energy used for centrifugation is four times the energy produced by the subsequent algal biodiesel product (Milledge and Heaven, 2013). To improve energy efficiency, the culture is pre-concentrated to 0.5% of the dry weight through a series of separation techniques. Another recommendation includes utilizing the entire biomass for energy production instead of only the lipid fraction (Amaro et al., 2017). Furthermore, 90% of energy consumption by the disc stack centrifuge can be reduced by optimizing three main factors: particle size, the rotational speed, and the outer radius of the centrifuge bucket (Abu-Shamleh and Najjar, 2020).

The decanter centrifuge consists of a horizontal conical bowl with a screw conveyor that rotates at high speeds to separate particles based on weight (Show et al., 2013). It was designed to handle high solid concentrations of up to 22% but necessitates massive energy utilization (Mohn, 1980). The multi-chamber centrifuge is made up of tubular bowls positioned coaxially to accumulate particles depending on the sizes in each chamber. This centrifuge design can separate up to 20% of solid concentration. However, the multi-chamber centrifuge requires manual cleaning of solids that is tedious, making it impractical for large-scale harvesting (Weatherley, 2013). The hydrocyclones is a cylindrical section attached to a conical base where microalgae culture is introduced from the top and cells move to the bottom in a cyclonic manner. Finer particles are discharged through the overflow pipe while larger particles are removed through the underflow (Show and Lee, 2014). Hydrocyclones can only handle low solid concentrations with low harvesting efficiency for particles of less than 400 μm in diameter (Gregg et al., 2009).

Overall, centrifugation comes with drawbacks that are time-consuming with high energy utilization as well as high capital and maintenance costs (Dassey and Theegala, 2013). Additionally, high gravitational force during centrifugation is physically damaging to cells which subsequently lowers yield for microalgae with delicate cell walls (Heasman et al., 2000). While centrifugation is effective for high-value products, the costs outweigh the yield for low-value products (Monte et al., 2018). Furthermore, centrifugation is unfeasible for saline environment usage as the high maintenance requirements would add to the already high cost (Najjar and Abu-Shamleh, 2020). Consequently, laboratory centrifugation was deemed more appropriate for concentrations of biomass over 30 mg·L^−1^ (Horowitz, 1986).

#### 5.2.2 Filtration

During filtration, microalgae are passed through a semipermeable membrane via gravity, pressure or vacuum force that strains microalgae cells from liquid media; extracting algal biomass (Amaro et al., 2017). This chemical-free technique can sieve through large amounts of cells with little physical damage to cells (Wicaksana et al., 2012). Conversely, filters are prone to rapid fouling and clogging which lowers throughput and increases maintenance costs (Milledge and Heaven, 2013).

Filtration under high pressure or vacuum is ideal to separate microalgae strains that are large such as *A. platensis* but is ineffective in recovering species that are smaller than 10 μm, for instance, *Dunaliella* and *Chlorella* (Hulatt et al., 2017). The pore size of the membrane determines the type of filter: micro-filters have pores larger than 10 μm, micro-filters range between 0.1 and 10 μm, ultrafilters have sizes of 0.02–0.2 μm, and reverse osmosis filter pores are smaller than 0.001 μm while tangential flow filters, vacuum filters, and pressure filters are few examples of relatively new filtration methods (Milledge and Heaven, 2013).

Most common microalgae are between the sizes of 5–6 μm (Edzwald, 1993), making micro-filters the most suitable membrane. Tangential flow and pressure filtrations are energy-saving techniques, providing output with higher energy than the initial energy consumed during the dewatering process (Danquah et al., 2009). Tangential flow filtration has a removal efficiency of up to 89% (Ma et al., 2014). On the other hand, ultrafiltration is suitable for long-term harvesting with better flux over time and fouling resistance compared to conventional micro-filtration (De Baerdemaeker et al., 2013). However, ultrafiltration has low energy efficiency and is expensive with high operating and maintenance costs (Ma et al., 2014). Belt filtration is successful in the water treatment industry especially for the separation of *Arthrospira* with low overall costs (Mohn, 1980). A novel electrochemical membrane filtration method was also successful in removing microorganisms and biomass separation. In addition, this technique was also shown to degrade pollutants and enhance membrane defouling better than hydraulic or backwash approaches (Merchant et al., 2007).

Membrane fouling is the most significant issue faced in the filtration process. Organic matter from algae cells tends to deposit onto the membrane resulting in a thick cake layer (Marbelia et al., 2016). Hydrophilic membranes are more resistant to fouling than hydrophobic membranes (Sun et al., 2014). Backwashing with subsequent forward flushing for 20 min was most effective in removing fouling in ultrafilters while sodium hydroxide (0.02 N) and sodium hypochlorite (100 mg·L^−1^) was applied to maximize flux recovery (Liang et al., 2008). *In situ* pre-oxidation method creates a porous and loose cake layer that increases flux but comes with extreme cell breakage and more release of organic matter (Qu et al., 2015). This can be mitigated by employing immobilizing catalysts to confine oxidation within the membrane interface. For instance, zero-valent iron nanoparticles were adhered to the membrane to activate peroxymonosulfate that oxidizes organic matter (Huang et al., 2020). The use of oxidation techniques also allows permeates to be recycled, reducing microalgae cultivation costs as well as water footprint. *D. salina* permeate recovered from ultrafiltration was successfully recycled after treatment with ultraviolet radiation and hydrogen peroxide (Guarnieri et al., 2018).

#### 5.2.3 Flocculation

The negative charge on microalgae surfaces prevents cells from self-aggregating which complicates the harvesting process. Flocculation uses organic and inorganic flocculants that neutralize this negative charge and promotes the accumulation of the microalgal cells. Flocculants carrying positive charge are supplemented to algae culture to absorb the negative charge on cell surfaces. This removes the electrostatic repulsion between cell particles and cells start to coagulate. Three critical aspects affect flocculation efficiency: surface charge neutralization, adsorption, and adsorption bridging (Polle et al., 2017). This approach is an appropriate harvesting technique for large-scale microalgae harvesting of a wide range of microalgae species (Hulatt et al., 2017). Flocculation is employed as the initial procedure in concentrating dilute suspensions of 0.5 g·L^−1^ of dry matter up to 100 times to a concentrate of 50 g·L^−1^. Subsequent mechanical harvesting techniques such as centrifugation will then result in an algal paste with 25% of dry biomass (Wileman et al., 2012). This combination makes the total energy utilization acceptable as the particles have agglomerated into large sizes, thus less processing water volume is required (Schlesinger et al., 2012).

Chemical flocculation has a low cost with easily obtainable chemical flocculants such as alum and ferric chloride (Bracharz et al., 2018). Metal salts yield separation of up to 95% of microalgal biomass but tend to remain in the biomass residue (Chatsungnoen and Chisti, 2016). These multivalent salts are influenced by their electronegativity and solubility; the more electronegative the ion, the faster the coagulation (Barros et al., 2015). Such chemicals are also highly toxic to the environment and thus require an additional removal treatment step which increases production cost (Uduman et al., 2010). Moreover, inorganic and synthetic flocculants have serious implications on human health including Alzheimer’s disease and other neurodegenerative disorders (Kumar et al., 2019b). As such, an extra pretreatment step is required to remove chemical residues from the harvested biomass. On the other hand, positively charged biopolymers such as chitosan are safer alternatives but only function at low pH, limiting this flocculation method to only acidic dwelling microalgae (Chang and Lee, 2012). A novel time-saving, economical, and scalable chemical flocculation method using potash alum at pH lower than 8.5 or with the addition of hydrochloric acid for cultures of pH over 8.5 was found to have a harvesting efficiency of up to 98.7%. This rapid chemical flocculation with multiple recycling lowered harvesting costs as low as $0.06 per kg of dry algal biomass compared to the typical harvesting cost of $3.3 (Mehta and Chakraborty, 2021).

In auto-flocculation, microalgae flocculate due to environmental stress such as nitrogen fluctuation, changes in pH, or photosynthetic CO_2_ depletion (Schenk et al., 2008). This process utilizes natural gravity settling that is inexpensive and less damaging to cells compared to centrifugation. Increasing pH in the presence of calcium and magnesium ions induces this phenomenon to yield high biomass recovery with over 90% efficiency (García-Pérez et al., 2014). This process is aided by the addition of 1M sodium hydroxide (Singh and Patidar, 2018). However, this natural occurrence only occurs in certain microalgae and is known to be slow and unreliable. Additionally, the use of sodium hydroxide is undesirable due to the tedious process control and alteration of cell composition (Schenk et al., 2008).

Bioflocculation is another safer and eco-friendly alternative to chemical flocculation. Bioflocculants are produced by microalgae, for instance, the external polysaccharides (EPS) of bacteria, certain microalgae, and fungi are used to flocculate algae in suspension (Hulatt et al., 2017). Bacteria with flocculant EPS are typically added to the microalgae culture supplemented with a suitable organic carbon source to prevent altering biomass productivity (Udayan et al., 2022). As this is a chemical-free process, there is no need for pre-treatment for the recovered biomass. For instance, the bacteria *Solibacillus silvestris* harvested *N. oceanica* at 88% efficiency while the fungi *Aspergillus oryzae* flocculated *C. vulgaris* with a removal efficiency of over 97% (Wan et al., 2013; Zhou et al., 2013). A novel polymeric bioflocculant from *Streptomyces* has also been identified. Using 0.5% of this bioflocculant (ABF), the flocculation rate was 99.18% within 10 min on *Nannochloropsis* (Sivasankar et al., 2020).

#### 5.2.4 Flotation

Flotation takes advantage of air or gas bubbles that adhere to microalgae cells to carry the suspended cells to the surface of the liquid media for harvesting (Laamanen et al., 2016). This technique has a simple operating procedure, a relatively high harvesting efficiency for both marine and freshwater microalgae as well as high processing throughput while being economical (Ndikubwimana et al., 2016). Furthermore, flotation can be a rapid process for certain microalgae species with low density and self-floating properties (Edzwald, 1993). Flotation often necessitates flocculants and is therefore used in tandem with flocculation (Rubio et al., 2002).

Dissolved air flotation (DAF) produces air bubbles by saturating the culture with compressed air before releasing the culture at high pressure (Edzwald, 1993). DAF utilizes minute bubbles of sizes between 10 and 100 µm (Edzwald, 1993). Chemical flocculation often precedes DAF to produce pure effluents. DAF boosts up to 95% removal efficiency when using surface-modified bubbles with cationic polymer (Henderson et al., 2009). A novel dissolved air flotation process that utilizes positively charged bubbles (PosiDAF) supplemented with the algal organic matter has a cell separation of over 90% (Rao et al., 2018). Regardless of DAF’s efficiency, it is still obstructed by high energy requirements due to the required high pressures and usage of chemicals (Hanotu et al., 2012).

Dispersed air flotation (DiAF) employs a sparger to continuously produce air bubbles of sizes 700 to 1,500 µm (Alhattab and Brooks, 2017). This technique has lower energy demand at the cost of expensive equipment and high-pressure drop for producing bubbles (Shin et al., 2016). Natural and synthetic collectors, for instance sodium dodecylsulfate, cetyl trimethylammonium bromide (CTAB), saponin, and chitosan have been used to support DiAF with high algal removal efficiency (Kurniawati et al., 2014). A surfactant-aided DiAF for *Chlorella saccharophila* and the CTAB had a recovery efficiency of 95% (Alhattab and Brooks, 2020). Marine microalgae have also been successfully harvested with a 23-fold rise in algal concentration and more than 99% recovery efficiency using an advanced flotation machine with dodecyl pyridinium chloride (Garg et al., 2014).

In electro-flotation, electrolysis generates microbubbles from electrodes to capture microalgae cells (Baierle et al., 2015). This method is chemical-free and can be applied to most microalgae species. It also simultaneously disrupts cells, enables recycling of culture media, has low process time, and continuous operation (Baierle et al., 2015; Krishnamoorthy et al., 2021). However, it is heavy on energy consumption with the need to frequently replace electrodes due to fouling (Singh and Patidar, 2018). A recent electrochemical dewatering approach was proposed using boron-doped diamond and aluminium electrodes for harvesting *Scenedesmus quadricauda* (Ryu et al., 2018). This technique that induces bioaggregation by combining floc-forming microorganisms and microalgae is an effective substitute for chemical flocculation, as it reduces toxic metal coagulant pollutants produced from electrochemical harvesting. Another low-cost novel electro-flotation design employs an Arduino-based magnetic stirrer, wherein a short distance between electrodes, medium mixing rates of 200 rpm with 50 W could decant as much as 100% of algal biomass from 500 ml of media (Sanchez-Galvis et al., 2020).

### 5.3 Drying of Microalgal Biomass

Microalgal biomass obtained directly from dewatering is usually dried before subsequent downstream processes as the dry solid content is low (Starkenburg et al., 2017). Drying inhibits microbial spoilage, increases the shelf life of the biomass, and lessens the costs of packing, handling, transportation and storage of the microalgal biomass (Bennamoun et al., 2013). An ideal drying method should dehydrate cells while reducing as much deterioration as possible to the delicate microalgal cells. Moreover, the time taken and costs incurred for the drying process must also be taken into consideration (Hosseinizand et al., 2018).

Traditional solar drying is the most common and cost-saving method as it utilizes direct energy from the sun (Marles et al., 2011). Drying from solar energy does not alter the composition of the microalgal biomass, as such is ideal for downstream processing of PHAs. However, it requires long drying periods with large drying surface areas, in addition to difficult quality maintenance due to biomass degradation and a high risk of bacterial contamination in open conditions as well as overheating (Shin et al., 2016). Open solar drying was also found to decrease as much as 40% in polyunsaturated fatty acids from *Derbesia tenuissima* biomass (Sizova et al., 2013). Closed solar drying systems that employ solar water heating systems have been developed that increase environmental temperatures to 60°C. Such drying methods can dry biomass to 10% water content within 5 h (Rochaix and Van Dillewijn, 1982). On the other hand, freeze-drying preserves cell constituents. The freeze-drying process was able to retain over 90% of protein composition (Shin et al., 2016). Regardless, this method aggregates more cells in crystal form resulting in a smaller cell surface area of contact with the extracting solvent, which would have significant effects on the cell wall integrity (Sizova et al., 2013). Spray drying is also utilized for many microalgae species (Hosseinizand et al., 2018). Here, atomized water droplets are sprayed into a vertical tower while hot gas is passed down and the dried biomass is collected from the tower base (Soeder, 1980). While drying is achieved within seconds, high pressure from the atomization procedure could damage cells and cause degradation (Show et al., 2019). Due to this atomization process, spray drying requires high energy demand and high capital (Chen et al., 2015).

Regardless of the many drying techniques available, the mechanism of how remaining water in the microalgal biomass affects nutrient extraction such as carbohydrates is not fully understood. There is much debate on whether this step is necessary. Water creates a barrier that prevents the effective nutrient mass transfer from the cells to the extraction solvent and is thus thought to be an indispensable process (Amaro et al., 2017). On the other hand, another hypothesis claims that the presence of water in the biomass improves nutrient extraction efficiency and can be removed (Medina et al., 1998). Indeed, nutrient extraction such as carbohydrates and lipids from wet microalgal biomass devoid of drying has been fruitful (Vieira et al., 2021). Since the drying step requires at least 89% of energy demand and incurs up to 75% of total processing cost, eliminating this step could prove economical and time-saving for large-scale production of microalgal biomass (Taher et al., 2014).

## 6 Pretreatment Methods for Microalgal Biomass

The microalgal carbohydrates are mainly in the form of cellulose and soluble polysaccharides in the cell wall, and starch in the plastids, with low hemicellulose content and absence of lignin. These carbohydrates need to be converted into fermentable carbon sources prior to microbial fermentation (Chen et al., 2013; Khan et al., 2018; Saratale et al., 2018). There are different pretreatment methods that have been studied to break down the algae carbohydrates into simple sugars, which can be majorly classified into physical, chemical, and biological pretreatments as listed in Table 5. The physical method utilizes direct steps for cell breakage which poses little issues to the environment but is plagued with high production cost due to high energy consumption that off-balances energy recovered from the biomass. Conversely, the chemical and biological methods require low energy utilization. The chemical method improves cell disintegration at a faster pace with lower energy demand but also comes with a high risk of chemical contamination towards the resulting biomass as well as the environment. While enzymatic pretreatments are more environmental-friendly with simple procedures without the need for complicated machinery, they incur high costs and long waiting periods. Therefore, the type of pretreatment chosen is important to efficiently convert these carbohydrates to sugars as the cell wall compositions vary according to microalgal species (Sankaran et al., 2020; Constantino et al., 2021; Debnath et al., 2021; Sirohi et al., 2021).

**TABLE 5:** Pretreatment methods for microalgal biomass.

	Physical	Chemical	Biological
Objective	Alteration of particle size, surface area, polymerization degree, and crystallinity index	Hydrolysis of cell wall	Enzymatic hydrolysis of cell wall
Types	• Mechanical—sonication, grinding, bead milling, extrusion, high shear impaction, fluid agitation, and homogenization	• Alkaline	• Enzymes
	• Radiation—thermal energy and microwave	• Acid	• Hydrolytic microorganism
	• Electrical—pulsed electric field, continuous electric field, high voltage electric discharge (HVED)	• Ionic liquid	
		• Ozone gas	
Advantages	• Efficient for carbohydrate removal	• Low energy requirements	• Environmentally friendly
	• Rapid	• Rapid	• Can be performed at mild operational conditions
	• Does not require hazardous chemicals	• Low cost	• No requirement for sophisticated instruments
		• Easy scalability	
Disadvantages	• Unsuitable for large-scale process	• Accompanied by high temperatures	• Highest cost
	• High cost	• Generation of toxic intermediates which may inhibit downstream fermentation	• Longer time requirement
	• High energy requirements		• Frequently requires other prior pretreatments methods
	• May require additional steps		

Physical pretreatment involves either mechanical, radiation, or electrical techniques (Sankaran et al., 2020; Debnath et al., 2021; Sirohi et al., 2021). It was found that total reducing sugars concentration was significantly affected by temperature whereas the concentration only slightly increased upon increasing the sonication time as shown from the sonification of *Chlamydomonas mexicana* biomass. Hence, the optimum sonication conditions for *C. mexicana* were set at 50°C and 15 min, while taking into account the energy and time consumption, which released 7.4 wt% of total reducing sugars of dry cell weight (Eldalatony et al., 2016).

Chemical pretreatment is usually performed via alkaline, acid, and ionic liquids, coupled with other reaction conditions such as high temperature and pressure to hydrolyze the microalgal cell wall (Sankaran et al., 2020; Constantino et al., 2021; Debnath et al., 2021; Sirohi et al., 2021). Through hydrothermal acid pretreatment, 50 g·L^−1^ of lyophilized *Chlorella sorokiniana* and *C. reinhardtii* biomass were added separately into 4 vol% of sulfuric acid solution before being autoclaved at 121°C for 30 min, which resulted in an increase of reducing sugar yield to 7% and 1%, respectively (Constantino et al., 2021). The application of ozone pretreatment on a mixed microalgal biomass under increasing dosages of 0.25, 0.5, 1.0, 1.5, and 2.0 g of applied ozone/g of dry weight biomass, without supplementary enzymatic hydrolysis led to microalgal cell breakage. However, the glucose conversion yields were still insignificant at less than 0.5 wt% of total carbohydrate (Keris-Sen and Gurol, 2017).

The most commonly used biological pretreatment employ the use of enzymes and hydrolytic microorganism, which can be microbes or fungi (Sankaran et al., 2020; Debnath et al., 2021; Sirohi et al., 2021). Occasionally, the microalgal biomass is pretreated by means of either physical or chemical methods prior to using this strategy (Sankaran et al., 2020). Enzymatic hydrolysis of *C. mexicana* performed using cellulase from *Trichoderma reesei* after sonication improved the yield to 28.05 wt% of total reducing sugars of dry cell weight. The previous sonication step partially hydrolysed the microalgal biomass, making it more susceptible to enzymatic hydrolysis (Eldalatony et al., 2016). Apart from physical pretreatment, chemical pretreatment can also partially disintegrate both the *C. sorokiniana* and *C. reinhardtii* biomass, resulting in higher yields of reducing sugar, from 7% to 1%, to 47% and 25%, respectively, when further two-step enzymatic saccharification was conducted on the chemically pretreated biomass using amyloglucosidase (600 U·g^−1^ biomass) (Constantino et al., 2021). Therefore, a combination of pretreatment methods can improve the release of reducing sugars.

## 7 In a Nutshell: Future Prospects and Conclusion

**FIGURE 2:**
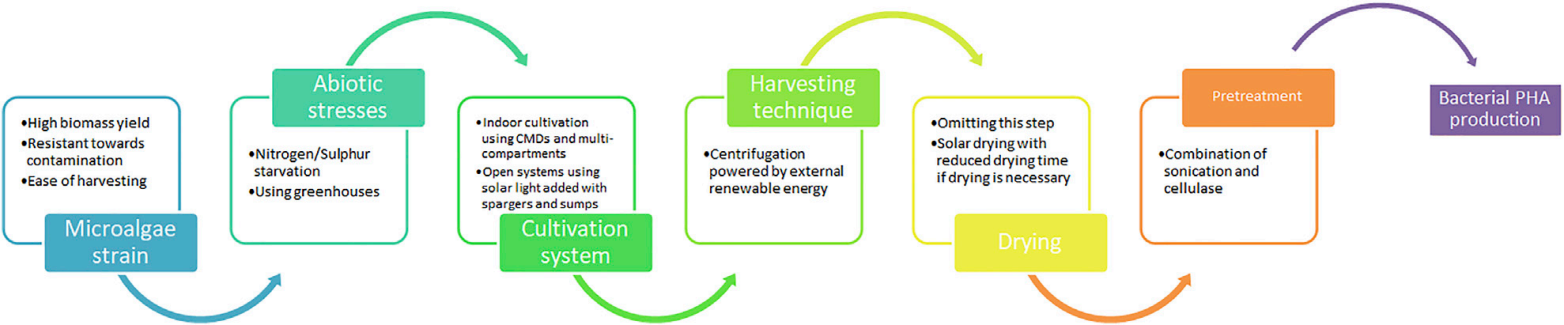
A model biorefinery process chain focusing on reducing energy requirement, economical cost. and maximizing biomass constituents.

Current microalgae productions only focus on an individual area such as emphasizing energy yield or boosting the value of the resultant bioproducts. To further ensure that microalgal biomass is able to compete with other carbon feedstocks, model biorefinery outlines focusing on reducing energy requirement, economical cost, and maximizing biomass constituents have been proposed (Figure 2). A biorefinery is termed as “an establishment that assimilates biomass conversion operations and equipment to yield fuels, power, and chemicals from biomass” (Jiang et al., 2016). The microalgae biorefinery system layout should take into consideration local conditions, regional climate, economics, infrastructure, and available resources. For instance, as many countries implement their own COVID-associated economic border restrictions, many sectors including the logistics industry have faced negative impacts.

The initial and most crucial step is to identify the strain of microalgae, either through species screening or genetic manipulations, that not only provides consistently high yields but also other necessary traits beneficial to the microalgal biomass manufacturing technology. For example, most natural microalgae cannot endure long-term open pond cultivation due to the high risk to contamination by fast-growing microbes (Assunção and Malcata, 2020). Introduction of genetically engineered microalgae with the ability to utilize limited nutrients and resistance towards field contamination could be an option.

As seen in Chapter 4, abiotic stresses can be utilized to modulate microalgal metabolite profiles and nutrients from the subsequent biomass attained depending on the desired end products. Nitrogen or phosphorus limitation has been the most efficient stress to amass high levels of carbohydrates. Similarly, for indoor cultivation of microalgae, usage of CMDs to increase CO_2_ utilization efficiency as well as a multi-compartment with different light acclimatation will increase productivity rates. Open systems are more economical with the advantage of using solar light added with spargers and sumps to disperse CO_2_. Both indoor and outdoor systems would benefit from using greenhouses for microalgae cultivation throughout the year to stabilize temperature.

On the other hand, there is no single harvesting technique that is compatible with all types of microalgae. While flocculation and coagulants are inexpensive and effective, the contaminants reduce the quality and quantity of the recovered biomass. Conversely, physical harvesting necessitates high operating cost and time which is uneconomical. Further research is required for more economical and eco-friendly optimum extractions. A suggested method is to reconstruct physical harvesting to be powered on external renewable energy such as solar panels or wind grids to offset the high energy requirement and decrease costs. Similarly, while it is possible to forgo the drying step, it is eminent for solar drying to be coupled with technology that reduces the drying time without sacrificing biomass quality in the event that drying is exigent.

As proven, the microalgal biomass manufacturing industry has made big strides to overcome obstacles that are bottlenecks for other feedstock production. Microalgae cultivation is possible with low-cost, energy-efficient RWP and PBR designs while economical harvesting techniques with high yields are available for large scale production. The flexible nature of microalgae to dwell in both freshwater and saltwater environments is advantageous for intensive algae production systems. On top of that, fertile land is unnecessary for microalgae growth and they can thrive even in industrial effluents; therefore, not only is there no competition for food production resources (Duffy et al., 2009), microalgae have also been applied as wastewater treatment (Liu et al., 2020). In regards to this, emerging studies have suggested cultivating microalgae in wastewater without nutrient supplementation which eliminates the need for media preparation (Karemore and Sen, 2015; Daneshvar et al., 2018). Additionally, industrial flue gas has been successfully utilized as carbon source for microalgal biomass production (Gentili, 2014).

Bacterial PHA production from microalgal biomass is a concept that is amassing more attention globally (Mishra et al., 2014). Research on the types of polymers that is obtained from microalgal biomass is needed in efforts to reduce single-use plastics. With the current post-COVID economy shifting towards sustainable energy, microalgae offer photosynthetic biomass for bacterial PHA synthesis as a substitute renewable feedstock which can be cultivated with limited natural resources with the extra benefits of phycoremediation besides CO_2_ sequestration.
